# General anesthesia is not superior to sedation in clinical outcome and cost‐effectiveness for ablation of persistent atrial fibrillation

**DOI:** 10.1002/clc.23528

**Published:** 2020-12-29

**Authors:** Zhengyan Wang, Lihong Jia, Tieying Shi, Changli Liu

**Affiliations:** ^1^ Cardiology Department The First Affiliated Hospital of Dalian Medical University Dalian Liaoning China

**Keywords:** atrial fibrillation, catheter ablation, cost, general anesthesia

## Abstract

**Background:**

The strategy of anesthesia used during ablation of atrial fibrillation (AF) remains controversial. This study aimed to compare sedation with general anesthesia (GA) for catheter ablation of AF.

**Hypothesis:**

The presence of AF is associated with an increased risk of stroke and heart failure and decreased quality of life and survival.

**Methods:**

We carried out a retrospective single‐centered study with 351 patients undergoing the first ablation procedure for AF under sedation or GA. The main outcome was freedom from recurrence of AF at 1 year. The total time of staying at the ablation laboratory and procedure cost were also calculated.

**Results:**

Freedom from atrial arrhythmia and ablation time did not differ between AF patients under sedation and GA (77.9% vs 79.9% and 42.27 ± 9.84 minutes vs 41.51 ± 9.27 minutes, respectively), while the total procedure time and cost were lower in patients who underwent sedation than GA (171.39 ± 45.09 minutes vs 202.92 ± 43.85 and 8.00 ± 7.02 CNY vs 8.79 ± 11.63 CNY, respectively).

**Conclusion:**

GA is not superior to sedation, in terms of ablation time and freedom from atrial arrhythmia at 1 year, whereas patients with GA had more anesthesia time and procedure cost than sedation.

**Abbreviations**
GA: General anesthesia
AF: Atrial fibrillation

## 1 INTRODUCTION

Atrial fibrillation (AF) is the most common human arrhythmia, affecting approximately 3% of the adult population and almost 6% of persons older than 65 years.^1^ The presence of AF is associated with an increased risk of stroke and heart failure and decreased quality of life and survival.^2^ The treatment of AF aims to decrease the risk of stroke (by anticoagulation when certain risk criteria are met) and improve the quality of life, either by preventing recurrences (the 'zrhythm control' strategy) or by controlling the heart rate during AF (the 'rate control' strategy).^3^, ^4^ Due to the severe limitations and low effectiveness of antiarrhythmic drug therapy in preventing AF recurrences, invasive methods including catheter ablation has gained widespread use in abolishing AF.^5^ In the last 20 years, catheter‐based ablation techniques have proved more successful in achieving rhythm control in AF patients.^6^

This procedure can be performed either under general anesthesia (GA) or under local anesthesia (LA) with sedation that may be conscious or deep. Anecdotally, the use of GA provides several potential advantages like greater comfort for the patients who are in a procedure lasting for a long time. Till now, even though some studies have demonstrated that GA is superior to LA for AF ablation in terms of arrhythmia recurrence and of the need for repeat ablation,^7^, ^8^ other organizations reported that for AF ablation, procedures under LA have similar results to GA regarding efficacy and safety after 1‐year follow up.^9^, ^10^ In addition, Martin et al found that there was a significant decrease in the number of GA patients being listed for repeat procedures, which means that it is also economically more effective.^8^

This retrospective, observational study aimed to estimate the clinical outcome, the related time and to calculate the cost‐effectiveness of both strategies.

## 2 METHODS

### 2.1 Patient population

This was a single‐centered retrospective study. We analyzed 351 consecutive patients with symptomatic AF who underwent the first AF ablation at The First Affiliated Hospital of Dalian Medical University between January 2014 and December 2015.

Patients underwent ablation either under conscious sedation with fentanyl and midazolam or under GA. In the GA group, after initiation of appropriate monitoring, induction of anesthesia was received using propofol 2 to 3 mg/kg and remifentanil infusion 0.02 to 0.3 μg/kg/min. The airway was secured either by endotracheal intubation (facilitated by administering a non‐depolarizing muscle relaxant) or insertion of a laryngeal mask airway. Anesthesia was maintained by continuous infusion of propofol and remifentanil, guided by vital signs and the use of a bispectral index (BIS) monitor.

### 2.2 Ablation strategy and time definition

The ablation strategy of paroxysmal atrial fibrillation was electrical isolation of pulmonary vein; the ablation strategy of persistent atrial fibrillation was electrical isolation of pulmonary vein and linear ablation; the ablation time was defined as the time from the beginning of ablation to the completion of the whole ablation strategy; anesthesia time was defined as the time from the patient entering the DSA operation bed to leaving the operation bed at the end of the operation.

### 2.3 Study outcome and total cost of the procedure

The primary study outcome was freedom from atrial arrhythmia lasting longer than 30 seconds after one ablation procedure, with or without the use of antiarrhythmic medications at 12 months and total cost of procedure defined as the expenses occurred in the catheter laboratory, including baseline AF ablation cost, anesthesia cost, and so on. The total cost was evaluated with the unit of Chinese yuan (CNY).

### 2.4 Follow‐up

Follow‐up was performed at 1, 3, 6, 9, and 12 months using a 12‐lead electrocardiogram at each visit. A 24 hours Holter monitoring was performed at 3, 6, 9, and 12 months. For nonparoxysmal AF patients, 7‐day Holter monitoring was performed at 6 months. A 3‐month blanking period was introduced after the ablation. Any atrial tachyarrhythmia lasting 30 seconds or more was considered a recurrence.

### 2.5 Statistical analysis

Continuous variables are presented as mean ± standard deviation and categorical data as counts or percentages. Analysis and comparisons of continuous data were performed using ANOVA, whilst the χ^2^ test was used to compare categorical data. A two‐sided probability level of <.05 was considered significant. All calculations were performed using SPSS 20.0 (IBM Software).

## 3 RESULTS

### 3.1 Patient population

A total of 351 patients were analyzed including 148 procedures with GA and 203 procedures with LA. Patient basic characteristics are summarized in Table 1. There were no significant differences between the two groups regarding most clinical characteristics, except that patients in the GA group had a longer time of AF.

**TABLE 1:** Basic characteristics of patients included in the study

Variables	Sedation (n = 203)	GA (n = 148)	*P* value
Age	61.98 ± 9.85	62.86 ± 8.75	.48
Male, n(%)	121(59.6)	87(59.20)	.68
Hypertension, n(%)	82(40.3)	63(42.5)	.16
Diabetes, n (%)	41(20.10)	32(21.6)	.11
coronary artery disease, n (%)	49(24.10)	35(23.6)	.31
Ejection fraction (%)	57.28 ± 3.65	55.62 ± 1.97	.13
Left atrial diameter, mm	37.56 ± 4.08	36.54 ± 4.69	.25
Time in atrial fibrillation, month	34.15 ± 6.78	44.59 ± 5.69	.01

### 3.2 The length of time spent in ablation laboratory

Table 2 compares the time‐length of anesthesia preparation and total procedure time. The length of time increased both for anesthesia preparation (GA 31.24 ± 6.68 vs Sedation 5.69 ± 1.96 minutes, *P* < .001) and total procedure (GA 202.92 ± 43.85 vs Sedation 171.39 ± 45.09 minutes, *P* < .001) in the GA group, while the ablation time did not show any difference between two sedation and GA groups (*P* = .17).

**TABLE 2:** Length of occupation time in catheter room

Variables	sedation (n = 203)	GA(n = 148)	*P* value
Preparation time of anesthesia (min)	5.69 ± 1.96	31.24 ± 6.68	<.001
Ablation time (min)	42.27 ± 9.84	41.51 ± 9.27	.17
Total time of procedure (min)	171.39 ± 45.09	202.92 ± 43.85	<.001

Abbreviation: GA, general anesthesia, SD, standard deviation.

*Note*: Data are presented as group mean (SD).

### 3.3 Outcome and cost‐effectiveness

During the 12 months follow‐up period, the proportion of patients who were freed of atrial arrhythmias in the sedation and GA group were 77.9% and 79.9%, respectively **(**Table 3 **)**. There was no significant difference between the two groups (*P* = .818).

**TABLE 3:** outcome and cost as a function of sedation and GA use

variables	Sedation (n = 203)	GA(n = 148)	*P* value
Freedom from atrial arrhythmias at 12 months (%)	77.9	79.9	.818
Total cost in catheter lab (ten thousand CNY)	8.00 ± 7.02	8.79 ± 11.63	<.001

Abbreviation: GA, general anesthesia.

On the other hand, the expenses of AF ablation in the GA group (87 900 CNY) were notably higher than those in the sedation group (80 000 CNY).

## 4 DISCUSSION

Radiofrequency catheter ablation of atrial fibrillation can be performed either under general anesthesia, or under conscious sedation with local anesthesia depending on the time, cost, and anesthesiology availability and /or center's protocol.^11^

General anesthesia provides sufficient control of pain and physical movement and stable respiration during AF ablation, which may facilitate durable AF and increases the success rate.^12^, ^13^ However, in addition to the cost, there is an incremental time for pre‐procedure patient evaluation by another group of physicians (anesthesiologist).^14^ We performed a retrospective study to evaluate the outcome of AF catheter ablation, time of anesthesia preparation, total time patents staying in the ablation laboratory, and the expenses of the procedure. We found that the primary outcome did not differ between the GA and sedation groups, while patients undergoing GA spend more time preparing for the anesthesia and staying in the catheter laboratory. Additionally, the cost of the total procedure rose in the GA group, compared with the sedation group.

There are previous studies investigated the differences in catheter ablation outcomes, but the results are inconsistent. Di Biase et al. presented that the use of general anesthesia is associated with a higher cure rate with a single procedure,^15^ while Bun and his coworkers found that for remote magnetic AF ablation, procedures under LA have similar results to GA in terms of efficacy and safety after 1‐year follow‐up.^9^ In our study, we did not find a significant difference in freedom from atrial arrhythmia lasting longer than 30 seconds, either. This inconsistency may partially be attributed to different AF population enrolled in studies. The study of Bun et al and ours included AF population with both paroxysmal and persistent AF, compared to Di Base's study only covered paroxysmal AF subjects. Martin CA et al.^8^ found that the success rate and economic benefits of general anesthesia, sedation, and ablation in patients with persistent AF were better. Unlike this study, more than 80% of patients in our study were paroxysmal AF patients. There were more linear ablation and fragmentary potential ablation in patients with persistent AF, and higher requirements for patients' stability and tolerance. Therefore, general anesthesia and sedation were more advantageous for patients with persistent AF. However, in the GA group, the cost of the procedure was higher and the total times that patients staying catheter laboratory was longer. Of note, the ablation time did not differ between the two groups, which mean that the anesthesia factor accounted for the increased time. However, some previous studies concluded similar data. For instance, Bun et al. and Malcolme‐lawes et al. also reported that ablation time between GA and sedation group did not differ.^9^, ^16^ But Staskova et al. found that the time needed for the preparation of patients was significantly longer in the GA group, while the time needed for electrical disconnection of all pulmonary veins was shortened.^17^ The reasons for these discrepancies between studies may come from the definitions of the times and basic clinical characteristics of patients included.^18^, ^19^

## 5 LIMITATIONS

This study was a single‐center retrospective study. The number of patients may not have been large enough to generalize the results. Furthermore, due to insufficient data, we could not analyze the details of time and cost in the ablation laboratory, and thus it is possible that some differences between groups were missed. Last, we did not evaluate other outcomes of patients such as the feeling of patients during or after the procedure, which could be an important factor in patients' whole life. The utility and potential advantages of general anesthesia may extend beyond the parameters measured in this study. For example, the avoidance of airway obstruction and potential air embolism when a patient draws a large negative intrathoracic pressure, and intrathoracic pressure during airway obstruction can be negative enough to overcome a hemostatic valve in a sheath with/without catheter or exchange wire. Further study with a larger sample size and more strict design was still needed.

## 6 CONCLUSIONS

For AF ablation, procedures under sedation had similar success rates and time of catheter ablation with that under GA, but the GA group had longer total time spent in the ablation laboratory and higher procedure cost. Nevertheless, how to choose the type of anesthesia can be assessed on a patient‐by‐patient basis. Additionally, operator and staff expertise is also the most likely determinant as to whether the deep sedation technique can be utilized by a particular laboratory.

## CONFLICT OF INTEREST

There are no potential conflicts of interest to disclose.

## AUTHOR CONTRIBUTIONS

Zhengyan Wang is responsible to the guarantor of integrity of the entire study, literature research, clinical studies, and manuscript preparation & editing; Lihong Jia is responsible to the study concepts, data acquisition, data analysis; Tieying Shi is responsible to the study design, statistical analysis, manuscript review; Changli Liu is responsible to the definition of intellectual content. All authors are approved in this manuscript.

## Data Availability

Not applicable.
